# Structural equation modeling for exploring the barriers to accessing healthcare among *hijra* in Bangladesh

**DOI:** 10.1371/journal.pone.0314478

**Published:** 2026-07-13

**Authors:** Mohammad Niaz Morshed Khan, Michiko Moriyama, Md. Enamul Haque, Syeda Naushin Parnini, Mohammad Abul Bashar Sarker, Shadat Hossain, Md. Masud Reza, Samira Dishti Irfan, Shehrin Shaila Mahmood, Gail Knudson, Sharful Islam Khan

**Affiliations:** 1 Graduate School of Biomedical and Health Sciences, Hiroshima University, Hiroshima, Japan; 2 International Centre for Diarrhoeal Disease Research, Bangladesh (icddr, b), Dhaka, Bangladesh; 3 Ministry of Health and Family Welfare, The Government of the People’s Republic of Bangladesh, Dhaka, Bangladesh; 4 Faculty of Medicine, University of British Columbia, Vancouver, British Columbia, Canada; PLOS: Public Library of Science, UNITED KINGDOM OF GREAT BRITAIN AND NORTHERN IRELAND

## Abstract

**Introduction:**

*Hijra* in Bangladesh and other South Asian countries face considerable barriers to healthcare given their potential exposure to repeated, multi-level stigmatization and marginalization. However, these needs remain unaddressed in the healthcare system, thus warranting exploration of their healthcare access barriers. This article examines healthcare access barriers among *hijra* in Bangladesh.

**Methods:**

A cross-sectional survey was conducted from June to November 2021 among 544 *hijra* across 16 districts out of 64 districts (25%) across all eight divisions in Bangladesh using multi-stage stratified cluster sampling, thus spanning a wide geographical coverage. Among all the participants, those who reported health problems in the last six months and sought treatment as a *hijra* (N = 215) were further used for analysis. Structural equation modeling (SEM) was utilized to assess and quantify the pathways how harassment, financial difficulties, discrimination, fear of discrimination, and lack of infrastructural facilities significantly creates barriers in accessing healthcare facilities among *hijra*. The results were expressed using factor loadings (FL) where FL closer to 1 indicated strong relationship.

**Results:**

The average age of *hijra* participants was 32 years old (±10.3 SD). The participants reported an average monthly income of US$107.2. Findings revealed that 53.5% of *hijra* respondents encountered four types of healthcare access barriers: harassment, discrimination, financial difficulties, and lack of infrastructural facilities in some form. Among these healthcare access barriers, our model illustrated that three were significant. These were discrimination by healthcare providers (Factor Loading = 1.14), which was the most significant barrier, followed by harassment by healthcare providers (Factor Loading = 0.93) and lack of infrastructural facilities (Factor loading = 0.41), and all these three barriers lead to an ultimate barrier to access to healthcare for the *hijra* community.

**Conclusion:**

This study highlights the critical barriers faced by *hijra* in accessing healthcare in Bangladesh. Findings suggest that interventions aimed at improving healthcare access should simultaneously address multiple factors to ensure gender-responsive healthcare systems that do not conform to a rigid gender binary.

## Introduction

### Global scenario of transgender populations’ health problems and healthcare-seeking behaviors

Transgender people, including transgender women, experience various forms of marginalization and social exclusion in many settings worldwide, including healthcare [1]. Notably, transgender people face health complexities due to biological, behavioral, psychological, and cultural diversities, compromised healthcare access, and their inclination towards self-gender reassignment [2,3]. For example, transgender people face higher risks of HIV and AIDS, diabetes, hypertension, mental health concerns and cancer compared to cisgender counterparts [4]. Specifically, a North American systematic review revealed that the prevalence rates of hyperlipidemia and hypertension both ranged up to 64.0% [5]. The same systematic review documented mood, anxiety and substance use disorders at 21.0%, 18.0% and 10.0%, respectively [5].

Global evidence highlighted ’cisgender populations’ healthcare access privileges [5], in contrast to barriers amongst transgender populations. Specifically, a study on transgender youth in Canada (N = 293, Oct 2013-May 2014) revealed that 47.0% did not receive necessary healthcare, and 61% cited anticipatory fears of negative healthcare provider experiences [6]. A US-based study (N = 27,715, Aug 2015-Sep 2015) showed that 33.0% of transgender respondents experienced at least one episode of verbal harassment, denial of services, or lack of healthcare provider knowledge [7].

### Healthcare access barriers: The regional context

Notably, adversities are pronounced amongst transgender populations in developing countries presenting socio-cultural, legal, economic, and religious barriers due to lack of culturally safe care. 17.6% of transgender people in Brazil (N = 626, Jul 2014-March 2015) reported that healthcare providers refused to discuss or address their health concerns [8]. Similarly, a study in Mumbai, India (N = 110, July-August 2014), indicated that only 1.5% of transgender women received public healthcare services, whereas 41.8% and 26.9% resorted to local pharmacies and traditional healers, respectively [9]. A study in Nepal (N = 30, Sept 2009) revealed that transgender women were refused services by clinicians, and encountered verbal abuse from healthcare providers and lack of informational confidentiality [10]. Likewise, a Pakistan-based study (N = 214, Jan-Oct 2020) highlighted that 81% and 79% of transgender women faced neglect and discrimination from healthcare providers [11].

### *Hijra* in Bangladesh: Brief background and context

In Bangladesh, the Government of Bangladesh recognized *hijra* as a separate gender category [12]. *Hijra* represent a community dynamic of leaders (*gurus*) with disciples (*chelas*) living in their residence and collecting money from marketplaces and other public settings as part of their traditional *hijra* occupation [13]. As most of them are assigned male at birth (though some are born intersex) but generally embrace particular feminine appearances, mannerisms and behaviors, they also fall under the transgender umbrella. *Hijra* may or may not be eunuchs (castrated) [13–15]. *Hijra* differ from a rare set of transgender women. Rather, this group of trans women do not partake in *hijragiri* and do not identify as part of the *hijra* sub-culture and also fall within the broader transgender umbrella [15]. These populations are beyond the scope of this study.

Despite legal recognition as a separate gender, *hijra* experience stigmatization, discrimination and compromised rights. This is attributed to gaps in understanding about gender, where societies and institutions perceive *hijra* gender attributes as unorthodox [16]. These circumstances constrained their healthcare access [13,17,18], as *hijra* in Bangladesh lack the sociopolitical space to pursue life with dignity, thus reducing their healthcare-seeking willingness [7]. This undermines universal health coverage (UHC), which underscores universal access to healthcare for all populations irrespective of socio-demographic status [19].

In Bangladesh and its immediate region, *hijra* are societally marginalized due to gender expression and identity. The conservative socio-cultural and legal frameworks instilled negative perceptions towards these populations. These barriers are rooted in punitive laws such as Section 377 of the Penal Code 1860, Section 74, Dhaka Metropolitan Police (DMP) Ordinance, 1976 [20]. This pertains to a colonized law which prohibits male-to-male sex and sex acts other than penis-in-vagina heterosex [20]. Due to societal assumptions about their anatomy and behaviors, they are also unfairly subjected to the same disciplinary measures as their MSM (men who have sex with men) counterparts. Thus, this law negatively affects their engagement with law enforcement and legal systems. These legal circumstances have also cultivated a stigmatizing environment for *hijra,* where they are unable to exercise basic rights, including healthcare access.

### Healthcare-seeking behaviors among *hijra*: Review of existing evidence

Qualitative evidence in Bangladesh depicted lived experiences of gender-based marginalization, stigmatization, and neglect within the family, societal, and healthcare settings among *hijra* [13,21]. A recent local review [16] revealed that *hijra* received sub-human treatment from culturally incongruent healthcare providers. Moreover, as most *hijra*’s decisions are governed by their *gurus* [13,16], this negatively affects their healthcare-seeking behaviors. Specifically, deviating from *guru*’s decisions could cultivate punitive ramifications such as a fine (*Don*) [22].

There is ample global literature about healthcare access barriers amongst transgender populations, yet limited research quantifies the magnitude of these barriers, particularly through structural equation modelling [23–25]. Most *hijra-*based research foregrounds HIV and STI knowledge and prevalence [26,27], with limited attention to multifaceted healthcare access barriers amongst *hijra* in this region. Previous studies were geographically narrow and adopted non-representative sample sizes, thus warranting research that spans across multiple districts with diverse *hijra* cultures, degrees of conservativity, and healthcare structures. Analysis also remains scant about different pathways, such as social and economic factors of healthcare access barriers [24,28]. Although it would be beneficial to explore these contexts through relevant theories. Since such theory-informed analyses remain limited, thus warranting relevant theoretical applications such as intersectionality and syndemic theory. These knowledge gaps aggregately impeded the scope to develop culturally competent health policies which could mitigate their healthcare access barriers. In this context, this study aimed to identify the factors contributing to healthcare access barriers among *hijra* in Bangladesh. To achieve this, structural equation modelling was utilized to test the hypothesis developed over the conceptual framework of healthcare access barriers, while quantifying the pathways contributing to the healthcare access barriers.

### Hypothesis

The present study aims to test the following two hypotheses:

Hypothesis of the second order construct:

**H**_**a0**_: Harassment, financial difficulties, discrimination, fear of discrimination, lack of infrastructural facilities, and legal and identity barriers, are not the barriers to accessing healthcare facilities among *hijra*.
**H**_**a1**_: Harassment, financial difficulties, discrimination, fear of discrimination, lack of infrastructural facilities, legal and identity barriers are the barriers to accessing healthcare facilities among *hijra*.

Additionally, these are the following hypotheses of path assumptions among the first order constructs:

**H**_**b0**_: Harassment, financial difficulties, discrimination, fear of discrimination, lack of infrastructural facilities, and legal and identity barriers has no significant interrelations among them.
**H**_**b1**_: Harassment, financial difficulties, discrimination, fear of discrimination, lack of infrastructural facilities, and legal and identity barriers has significant interrelations among them.

### Conceptual framework

**Fig 1:**
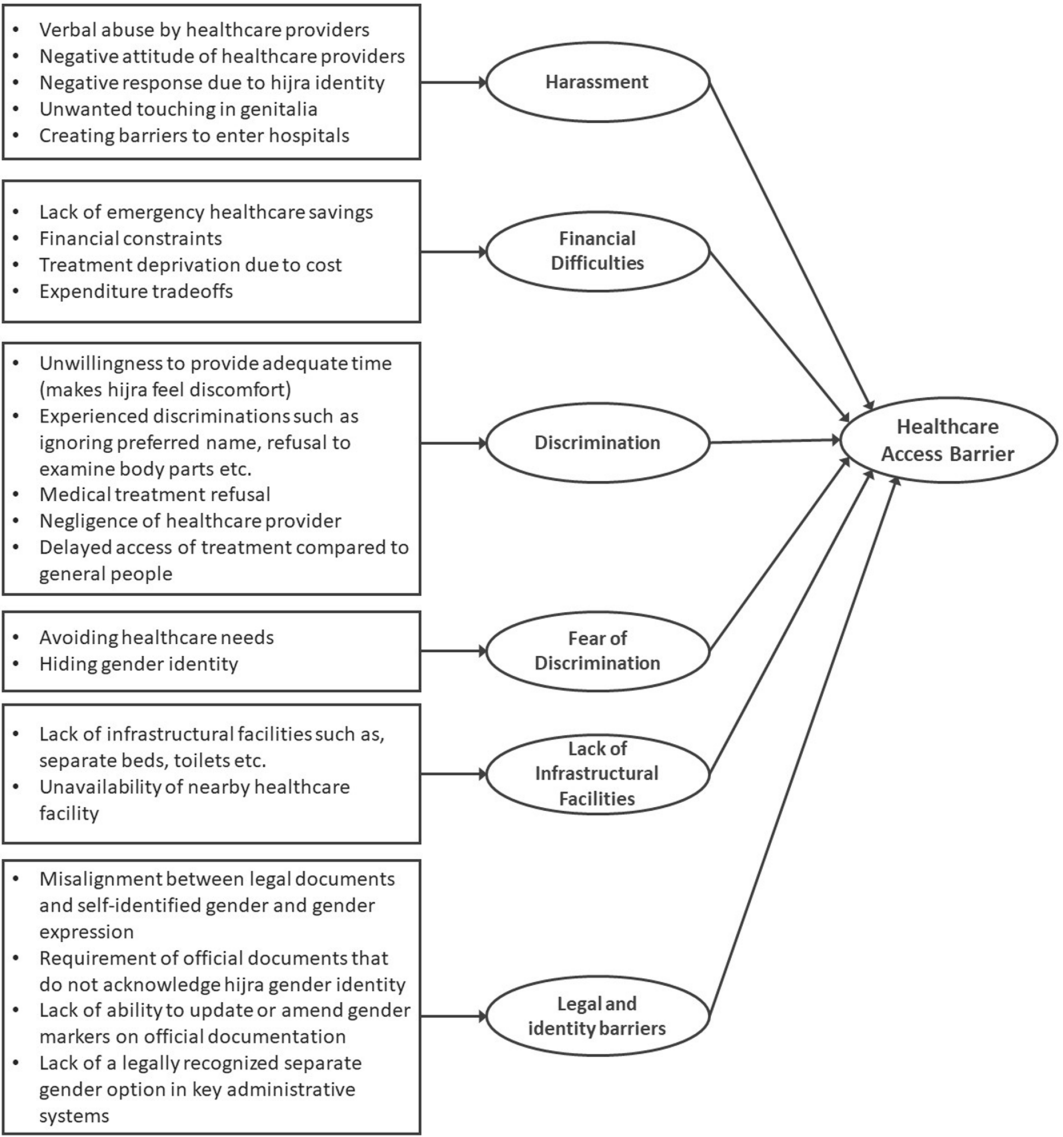
Conceptual framework of healthcare access barrier.

The conceptual framework is illustrated in **Fig 1**. Notably, this conceptual framework was adapted from Lodge et al.’s framework (2025), which synergized intersectionality and syndemic theory. While we retained the multi-domain structure of the access challenges faced by transgender populations, the conceptual framework applied to different outcomes and contexts. Nevertheless, we maintained its overarching structure to conceptualize healthcare access barriers among *hijra* in Bangladesh, and modified indicators and domains to reflect local socio-legal realities and the measurement needs of our structural equation model.

Notably, the WHO Health Systems Strengthening Glossary described healthcare access barriers as “the perceptions and experiences of people as to their ease in reaching health services or health facilities in terms of location, time and ease of approach” [29]. While location and time are beyond the scope of this research, several constituents of “ease of approach” are presented as indicators in our structural equation model. The literature coined these healthcare access constraints as the lack of culturally sensitized care, misbehaviors and neglect amongst healthcare providers, institutional barriers to entry, and financial limitations, which were all reflected in the conceptual framework.

In this context, *hijra*’s healthcare access barriers can be conceptualized as a composite of various barriers under six interrelated main themes, i.e., harassment, financial difficulties, discrimination, fear of discrimination (not lived discrimination, rather perceived and anticipatory discrimination), lack of infrastructural facilities, and legal and identity barriers. These themes, all classified as structural inequalities as per the modified intersectionality framework, determine healthcare access barriers for the *hijra* community. Moreover, as per the syndemic theory, these healthcare access barriers cluster and reinforce one another. In this context, harassment was defined as any type of verbal abuse by healthcare providers, negative attitude towards the *hijra* community, negative response due to *hijra* identity, sexual annoyance such as unwanted touching of genitalia, and impediments to entering the healthcare facilities.

**Fig 2:**
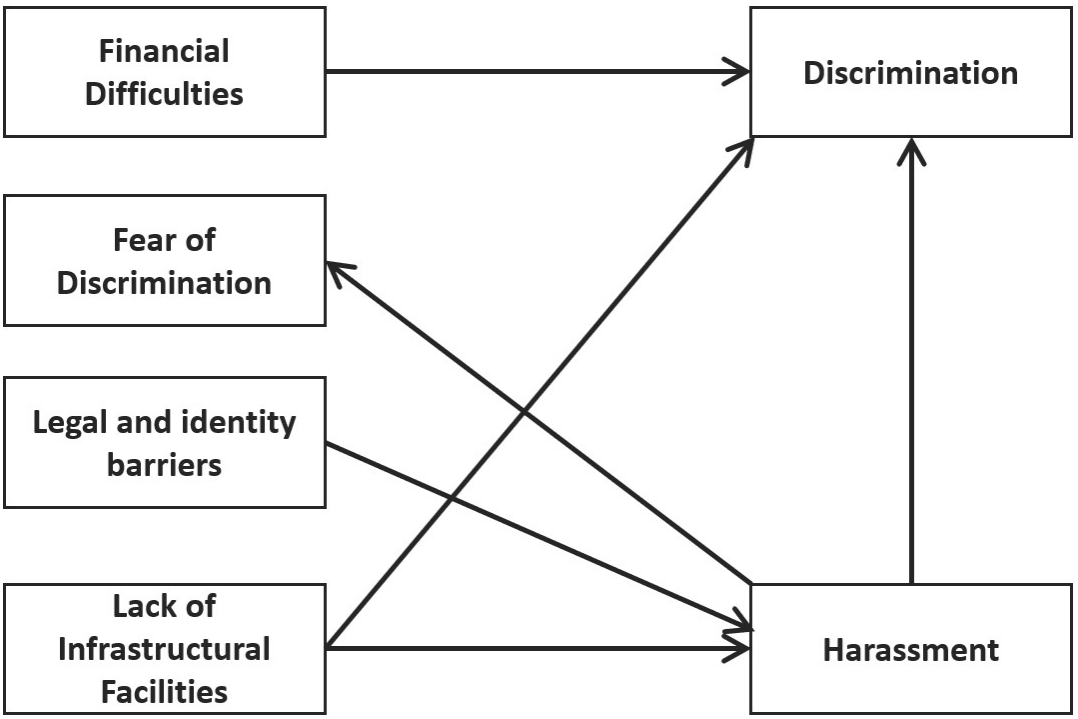
Conceptual interrelation framework among the first order latent constructs.

Financial difficulties included parameters such as lacking savings for emergency healthcare, financial constraints, being deprived of treatment due to cost, and expenditure tradeoffs, i.e., being unable to afford healthcare costs due to other living expenditures, etc. Discrimination included feelings of discomfort while disclosing healthcare problems due to judgmental responses, ignoring preferred names, refusing medical treatment or examination, negligence based on being *hijra*, delays in receiving necessary treatment, medications, appointments with doctors, etc., compared to the general population, etc. The fear of discrimination included: avoiding healthcare needs or hiding gender identity, etc. due to anticipatory fears. Lack of infrastructural facilities, such as separate beds and toilets for *hijra*, and unavailability of nearby healthcare facilities were termed lack of infrastructure. Furthermore, we conceptualized interrelations among the first-order latent constructs, reflecting influences between them (Fig 2). Within the framework in Fig 2, financial difficulties, harassment, and lack of infrastructural facilities was perceived to have a positive impact on discrimination. Also, lack of infrastructural facilities was perceived to affect harassment, and finally, harassment was perceived to affect the fear of discrimination. Another construct, i.e., legal and identity barriers, was defined as the mismatch between legal documents and self-identified gender; requirements of official identifications which do not acknowledge *hijra* gender identities; the lack of ability to update or amend gender markers on official documentation; and the lack of a legally recognized option for a separate gender in administrative systems.

Both of the abovementioned conceptual frameworks were developed through a combination of literature review, our prolonged programmatic and research experiences from working with *hijra* and community consultation with the *hijra* on what should be the key constituents of the conceptual framework [13,21,30–32]. Compared to Lodge et al.’s work, we changed the framework’s focal outcome to healthcare access barriers, and operationalized this outcome as a higher-order latent construct consisting of locally salient first-order domains (such as discrimination, harassment, financial constraints, fear of discrimination, infrastructural challenges, and legal and identity barriers). Moreover, we have indicated specific hypothesized interrelations among first-order constructs (Fig 2) to reflect how barriers could enforce each other (as per syndemic theory) through clinical encounters and administrative processes.

## Materials and methods

### Design

This cross-sectional study was conducted among *hijra* throughout 16 districts under all eight divisions in Bangladesh. This study opted for geographical diversity to ensure data representativeness and capture diverse perspectives. This study used the term “healthcare facility” to describe private and public healthcare facilities besides Non-Governmental Organization (NGO) clinics, where the healthcare facility typologies were explicitly mentioned.

### Theoretical framework: A syndemic and intersectional framework

Transgender populations, especially in structurally challenging settings, experience multilayered psychosocial and structural factors, e.g., stigma, poverty and substance use, which often cluster and interact with one another. This calls for the syndemic theory, which describes how historical, cultural and legal complexities affect health outcomes and healthcare-seeking behaviors [33]. Syndemic theory postulates that various constructs, such as discrimination and poverty, are interlinked with various co-occurring health conditions [34]. Although syndemic theory explains the interactions and clustering of psychosocial and contextual factors, it receives criticism for prioritizing biomedical factors. In this process, syndemic applications do not always describe how structural power and social positions contribute to unequal exposure to health conditions. Therefore, this analysis adopted an intersectional syndemic approach, where syndemics inform out focus on the synergy between multiple healthcare access barriers. Whereas, intersectionality illuminates the role of intersecting systems of oppression (stigma/discrimination, socioeconomic exclusion, and legal/identity governance) in creating patterned disadvantage [35].Intersectionality explores the interlocking effects of multiple forms of oppression predisposing individuals to unfair inequalities [36]. This proposition originated from Kimberle Crenshaw’s black feminist discourse on the intersecting roles of race and gender in exacerbating marginality. However, over the years, the concept has expanded to accommodate various forms of oppression rooted in age, religion, ethnicity, disability status, occupation, and gender identity/sexual orientation [37,38]. While intersectionality explains how intersecting social positions and structural power influence patterned exposure to multiple challenges, syndemic theory deliberates on how these barriers cluster and enforce one another, and cultivate healthcare access barriers. This study has accounted for intersectionality by treating barriers as structurally produced rather than as individual risks and examining how these barriers vary by key social positions, rather than assuming they are a homogeneous transgender population. In this analysis, discrimination, harassment, financial difficulties, infrastructural limitations, and legal and identity barriers are conceptualized as interacting and co-occurring conditions that cumulatively constitute healthcare access barriers. This aligns with the current structural equation model, which treats these domains as inter-related and co-producing the higher-order barrier construct.

### Sample size calculation

The sample size was calculated using Cochran’s formulae (please see Annex-1). To calculate the sample size, we reviewed literature on healthcare-seeking barriers among transgender populations worldwide and prepared a table. The sample size was calculated for each indicator. Finally, a maximum sample size of 575 was selected for *hijra* in 16 districts under 8 divisions, with 95% confidence intervals, a design effect of 1.5 (to adjust the clustering of observations), and a 5% level of precision. After adjusting for Finite Population Correction (FPC), the calculated sample size was 517. To compensate for drop-out during the interview or refusal to participate, the sample size was inflated by 5%. Therefore, the target sample size was 543, where 544 samples were finally collected. Annex 1 provides the detailed sample size calculation.

### Study participants and sampling strategies

To create the sampling frame that enabled the random sample of participants, a special mapping exercise was conducted in each district (including Dhaka) based on a *birit*-based method [26,39]. This approach was used in counting *hijra chelas* during the mapping exercise for each of the *gurus* and then proportionately distributed the target sample size according to the number of *chelas* in each *gurus* that was used in conducting HIV surveillance rounds in Dhaka in 2010, 2013, 2015, and 2020 [26,40]. A ‘*birit*’ refers to defined area where all *chela* activities are conducted. However, mapping methods were slightly modified due to time and resource constraints, particularly for Dhaka, since many *Gurus* live in the Dhaka district. To count *hijra chelas* in Dhaka, we prepared a list of *Gurus* in the first stage through a consultation meeting with *hijra,* then visited a randomly selected one-third of the *gurus.* For the second randomly chosen one-third of the *Gurus*, we called them via phone. For the remaining one-third, we used mapping data from the HIV surveillance 2015 (43) to extrapolate data for 2021.

For the in-person visits, we explained the objectives and benefits of the study. We asked them how many *chelas* were in their *birit* and recorded data using a prescribed format containing the sampling frame of *chela* for each *Guru* in a *birit*. We also visited all *chelas*’ houses and inquired about their availability for interview. For the second one-third, when we called *Gurus*, we explained the objectives and benefits of the study. Then we inquired about the number of *chelas* in their *birit* and recorded the data in the same prescribed format. For extrapolating the number of *hijra chelas* for the remaining one-third, we looked at the name of *Gurus* in the first and second part of the list in 2021 where we completed counting of *hijra chela* through visits and phone calls. Then we matched the name of *Gurus* between 2015 and 2021 to calculate the percentages of *hijra chelas* that increased in 2021 compared to 2015. Thereafter, we multiplied the number of *hijra chela* in 2015 with these percentages to extrapolate the number of *chela* in 2021, particularly for these *Gurus*. The calculation process is described in Annex-1. Thus, in 2021, the total number of estimated *hijra chelas* in Dhaka was 1,156. In total, from 16 districts, 1,156 + 2,544 = 3,700 *hijra chelas* were counted from 64 + 118 = 182 *hijra Gurus* in Bangladesh.

The study sample included 544 *hijra*. Quantitative surveying methods assessed healthcare access barriers among *hijra*. Using multi-stage stratified cluster sampling techniques, *hijra* were interviewed in each district [39]. In the first sampling stage, to ensure adequate representation of *hijra*, one district was initially selected from each administrative division with a city corporation. Thereafter, one district was chosen randomly from the remaining districts of each division by adopting the probability proportional to size (PPS) sampling technique [40]. In the second sampling stage, *hijra* were interviewed in each cluster (*hijra guru*’s *birit*, i.e., ritualized jurisdiction which *hijra* carry out for traditional *hijragiri*). The research team members and investigators work under the Global Fund project, operating an HIV prevention intervention at 50 service centers across the country in 37 out of 64 districts in Bangladesh. For this initiative, we solicited implementation support of two NGOs and community-based organizations (CBOs), who are tightly networked with these population groups. We have conducted consultations with the CBOs and *hijra gurus* to enlist their support. Additionally, *hijra* were recruited as research guides to facilitate access to their community. We also enlisted the support of *Gurus* to mobilize the participants, and ensured private spaces for conducting interviews. The interviews were held at the residence of the *hijra* or the *hijra guru* (leader), to maintain privacy and confidentiality. Before interviews, informed verbal consent was taken from *hijra*. All of these enabling factors have enabled us to achieve a 100% response rate.

Study participant recruitment started from 1 June 2021 and ended on 30 November 2021. Participants included those who identified themselves as belonging to the traditional *hijra* sub-culture, aged 18 years or older, and provided verbal informed and understood consent. The consent-taking episode was witnessed by the research guides who facilitated access to the *hijra* study participants.

The socio-demographic characteristics of 215 participants are illustrated in (**Table 1**). The mean age was 32.0 years. At the time of the survey, only 7.4% of *hijra* reported being married to a cisgender woman. Approximately 9.8% of *hijra* received no schooling, and 12.6% stated they could only sign their names. 74.4% of the participants had 7.1 years of schooling on average. The results showed that the majority of the participants’ occupation, i.e., the primary source of income in the last six months, was *Cholla khata,* i.e., collecting money from the shopkeepers (84.7%), followed by *Badhai khata,* i.e., money as gratuity for blessing newborns*/ Dholl dance* (75.3%), and sex work (34.9%), where their average income was 11602.6 Taka, or 107.2 USD (1 USD = 108.2 Taka; according to Bangladesh Bank, 15^th^ June, 2023) [50]. According to recent Integrated Bio-Behavioral Surveillance findings of 2020, the HIV prevalence of *hijra* under intervention coverage was 0.9%. [26] Whereas, our programmatic case detection data from the previous year was 0.1% amongst *hijra*. However, HIV prevalence data were not collected, as it was out of the scope of this study.

**Table 1:** Socio-demographic characteristics of the participants.

Variables	Frequency (%) N = 215 Unless otherwise stated
**Marital status**
Currently married	16 (7.4)
Currently unmarried ^†^	199 (92.6)
**Currently living with whom**
Alone	76 (35.4)
With non-*hijra* friend	2 (0.9)
With *hijra* friend	11 (5.1)
With parikh (Male lover)	57 (26.5)
With *hijra Guru*	41 (19.1)
With family	28 (13.0)
**Age (in years)**
18-24	58 (27.0)
25-34	76 (35.3)
35-44	51 (23.7)
45+	30 (14.0)
	Mean (SD) = 32.0 (±10.3) Median (IQR) = 30.0 (24.0–38.0)
**Years of schooling**
Illiterate/ Never been to school	21 (9.8)
Can sign only	27 (12.5)
Less than one year	7 (3.3)
1-17 years of schooling	N (%) = 160 (74.4) Mean (SD) = 7.1 (±3.0) Median (IQR) = 8.0 (5.0–9.0)
Main sources of income in the last six months*
Business	4 (1.9)
Service (Private/Govt)	4 (1.9)
Sex work	75 (34.9)
*Badhai khata*/ *Dholl* dance	162 (75.3)
Dance/Song (*Baina*)	14 (6.5)
*Cholla khata*	182 (84.7)
*Randhuni* (Cook)	5 (2.3)
Maid servant	1 (0.5)
Tailor	1 (0.5)
**Average income in a month within last six months (in Taka)**
≤10,000	106 (49.3)
10,001-20,000	93 (43.3)
>20,001	16 (7.4)
	Mean (SD) = 11602.6 (±6564.9) Median (IQR) = 10166.7 (7500.0–13708.3)

*Multiple responses; here, SD = Standard deviation, IQR = Inter quartile range.

† hijra who were widower, separated, and divorced were included in the currently unmarried category during the time of interview.

### Data collection

The study team consisted of a panel of multidisciplinary experts including an epidemiologist, sociologist and anthropologist. They are also worked alongside *hijra* community members who served as community research partners. Specifically, their role involved co-designing the data collection tools, guiding the development of the conceptual framework and supporting in field testing and data collection. A trained team performed data collection manually using printed questionnaires. Before data collection, both peer and non-peer researchers received week-long interactive and extensive training on data collection. Data were collected face-to-face using a validated, researcher-administered semi-structured questionnaire. This questionnaire was prepared and pretested in the field, and fine-tuned based on field-testing experiences.

### Measurement

#### Outcome.

The present study investigates healthcare access barriers, which are not directly observable variables. Instead, it is a second-order latent construct, where a second-order latent construct indicates a higher-order factor combining two or more first-order latent constructs to represent an abstract and overarching construct. To measure this second-order construct, the study adopts four distinct first-order latent constructs, i.e., harassment, discrimination, financial difficulties, and lack of infrastructural facilities. In this case, the first-order latent constructs were developed from different sets of observed variables (described in the following section).

#### Covariates.

Several single and multiple response variables were considered for constructing the observed variables of this study which were aligned with the study’s conceptual framework and availability of variables in the dataset. The design of these observed variables showed an increase in the level of obstacles or negative attributes with an increase in their respective values. The newly constructed observed variables were then used to formulate the four first-order latent constructs, i.e., harassment, discrimination, financial difficulties, and lack of infrastructural facilities, which aligned with the conceptual framework of this study [41]. Harassment was measured using six different observed variables, i.e., a) experience of verbal abuse in medical settings during their lifetime (no = 1, do not remember = 2, and yes = 3), b) negative attitude of the healthcare providers during last visit (very positive = 1, positive = 2, neither positive nor negative = 3, negative = 4, and very negative = 5), c) negative response from service providers at public healthcare facilities while revealing *hijra* identity (behaved well = 1, average behavior = 2, express reluctance/unwillingness to provide treatment = 3, and misbehaved = 4), d) entry barriers at public healthcare setting measured through three multiple response questions, i.e., barriers to entering the hospital gate, or standing in the queue, or entering name/purchasing ticket (the three multiple response variables were binary coded, where 1 = faced harassment, 0 = otherwise, and finally the sum of multiple responses ranging from 0 to 3, was used as the observed variable), e) entry barriers at private healthcare setting (similar to entry barriers at public healthcare setting, as a sum of multiple responses ranging from 0 to 3, was used as observed variable), and f) different types of harassment experienced at healthcare facilities measured through two multiple response questions, i.e., belittling/ridiculing because of *hijra* identity, or denying *hijra* identity (the two multiple response variables were binary coded, where 1 = faced harassment, 0 = otherwise, and finally the sum of multiple responses ranging from 0 to 2, was used as the observed variable).

Similarly, discrimination was observed using seven different observed variables. The observed variables included: a) negligence from public healthcare providers while seeking treatment (coded as a sum of multiple responses, i.e., healthcare providers listened to their problems sometimes = 1, didn’t listen at all = 2, asked about their problems sometimes = 1, didn’t ask at all = 2, ranging between 0–6, was used as the observed variable), b) negligence from private healthcare providers while seeking treatment (coded the same as negligence from public healthcare providers while seeking treatment, as a sum of multiple response ranging from 0 to 6, was used as the observed variable), c) refusal/ denial of treatment by the healthcare providers while seeking treatment during their lifetime (no = 1, yes = 2), d) different types of discrimination experienced at healthcare facilities during their lifetime measured through two multiple response questions, i.e., ignoring preferred name, or refusal to examine body parts (the two multiple response variables were binary coded where 1 = faced discrimination, 0 = otherwise, and finally the sum of multiple responses ranging from 0 to 2, was used as the observed variable), e) feelings of discomfort while sharing health problems with healthcare professionals at public healthcare facilities due to non-cooperative behaviors (very comfortable = 1, comfortable = 2, uncomfortable = 3, and very uncomfortable = 4), f) feelings of discomfort while sharing health problems with healthcare professionals at private healthcare facilities due to non-cooperative behaviors (coded same as feelings of discomfort while sharing health problems with healthcare professional at public healthcare facilities due to non-cooperative behaviors), and g) delayed access of treatment compared to general populations measured through six multiple response variables, i.e., delay while getting medicine, treatment intervention such as surgery or other procedure, diagnostic test, support from nurse, serial from primary healthcare, and obtaining serial from specialized healthcare setting in the last one year (the six multiple response variables were binary coded where 1 = faced discrimination, 0 = otherwise, and finally the sum of multiple responses ranging from 0 to 6, was used as the observed variable).

The construct, financial difficulties was measured using four observed variables, i.e., a) lack of emergency savings for healthcare (no = 1, yes = 2), b) financial difficulties to receive healthcare services ever in a lifetime (never = 1, very few times=2, sometimes = 3, and always = 4), c) necessary living expenditure tradeoff to seek healthcare services ever in a lifetime (no = 1, yes = 2), and d) disruption/ abstinence from treatment due to lack of money in the last year (no = 1, yes = 2).

Lack of infrastructural facilities was measured using three different observed variables, i.e., a) absence of convenient location of healthcare facilities to get health services (no need to go anywhere = 1, need to visit another area = 2, need to visit another city = 3, and need to visit another country = 4), b) lack of infrastructural facilities in public hospitals measured through two multiple response questions, i.e., beds in health facilities for *hijra*, or toilets in healthcare facilities for *hijra* (the two multiple response variables were binary coded where 1 = lack of infrastructural facilities, 0 = otherwise, and finally the sum of multiple responses ranging from 0 to 2, was used as the observed variable), and c) lack of infrastructural facilities in private hospitals (similar to lack of infrastructural facilities in public hospitals, as a sum of multiple responses ranging from 0 to 2). However, due to the sensitive nature of legal and identity-related issues, we could not collect relevant data from the participants for this construct (please see explanation in the discussion section).

### Data cleaning and preprocessing

The dataset contained a total of 544 observations. Initially, each respondent was asked whether they had health problems in the last six months. Among them, 429 (78.9%) reported health problems and 115 (21.1%) reported that they did not have health problems in the last six months (automatically excluded in the SEM analysis). Thereafter, 429 respondents who reported illness were asked whether they sought treatment as a *hijra* from private or government healthcare facilities. Among 429 respondents, 215 (50.1%) responded that they sought treatment from private or government healthcare facilities as a *hijra* whereas 214 (49.9%) did not seek treatment. Among those who did not seek treatment,184 (86.0%) did not feel the need to seek treatment and 30 (14.0%) did not seek treatment due to fear of discrimination. We have compared the socio-demographic characteristics among participants who reported health problems and sought treatment (N = 215) vs. those who had health problems but did not seek treatment (N = 214) and found no significant differences (Please see Annex-2). Therefore, these individuals (N = 214) had no data for further analysis. Finally, 215 observations from 15 different districts (except Jashore, where everyone reported had health problems but did not feel the need to seek treatment) were then used for analysis.

### Variable selection process

Variables were primarily selected upon extensive literature review and availability of variables in the survey dataset. At first, an all-in model was performed with primarily selected variables. Variables were then determined using the backward selection method [42]. The fear of discrimination latent construct was removed entirely as it only contained two items, which could not correctly reflect the construct. As previously mentioned, legal and identity barriers could not be considered as a construct as we did not collect data on this due to the sensitivity of this topic. In the variable selection process, items with the lowest factor loadings were eliminated in each step [41]. The model was re-run for every systematic backward elimination to check the updated situation and model fit. After repeating this process multiple times, variables, i.e., sexual abuse by public healthcare providers (by touching genitals), similar accounts of sexual abuse by private healthcare providers, avoiding healthcare facilities due to fear of discrimination, hiding *hijra* gender identity while visiting healthcare facilities, the distance to healthcare facilities from home in kilometers, and respondents’ opinion on the proximity of healthcare facilities (public/ private/ NGO/ others) near home were eliminated in the process. Finally, we attained a model with adequate fit. Twenty variables (six harassment, seven discrimination, three lack of infrastructural facilities, and four financial difficulties) were selected at the end and further used to describe the scenario of healthcare access barriers (illustrated in **Fig 4** in the results section).

### Constructing a composite variable for healthcare access barriers

**Fig 3:**
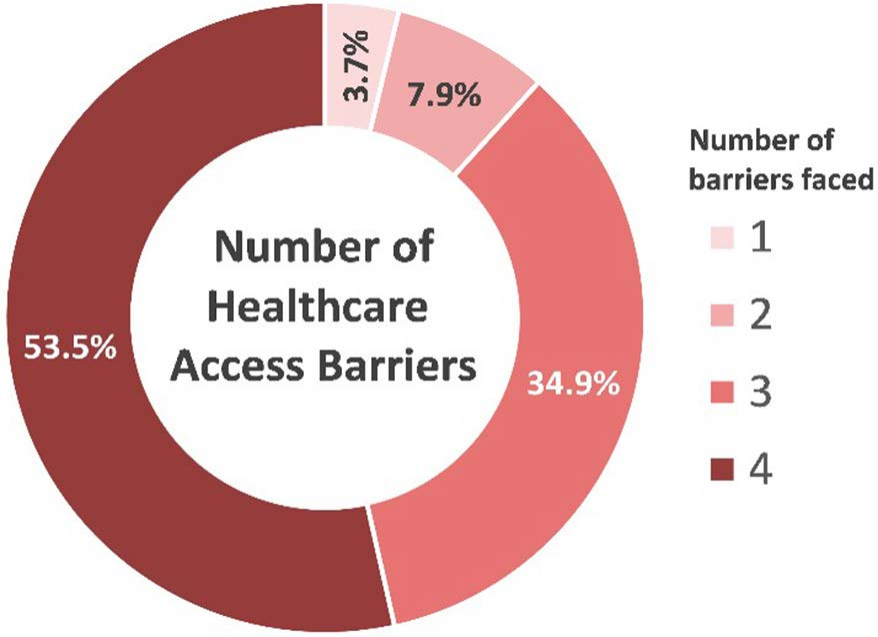
Percentage of *hijra* facing a different number of healthcare barriers (N = 215).

We followed a two-step composite variable-making process to achieve the ‘number of healthcare access barrier faced variable [43,44]. This was done to visualize and quantify the different number of barriers faced by the hijra to access healthcare in Bangladesh. The variable was considered as a composite score of different themes, rather a scale measure. The thematic areas were constructed with thorough literature review and programmatic expertise of the researchers, following a process similar to meaningful grouping [43,44]. Initially, twenty selected variables were transformed into respective binary responses of respondents either facing (1) or not facing (0) healthcare access barriers. The responses were then aggregated into four binary healthcare access barrier themes, i.e., harassment, financial difficulties, lack of infrastructural facilities, and discrimination. Finally, the four themes were aggregated to leverage the healthcare access barrier variable (see **Fig 3** in the results section).

### Statistical analysis

We formulated a conceptual framework to examine the interconnectedness between harassment, discrimination, lack of infrastructural facilities, and financial difficulties. The framework investigated the pathways leading to healthcare access barriers (see **Fig 1**). To validate the proposed hypothesis, we employed confirmatory factor analysis (CFA) within Structural Equation Modeling (SEM) to test whether the relationships between observed variables and their underlying latent constructs align with the conceptual framework [41]. In our study, as the dependent variable, i.e., healthcare access barrier was not directly measurable, rather a second order latent construct. Thus, traditional multivariate regression modeling was out of the scope of this study. Considering this, researchers prefer the SEM approach in these particular cases as it allows testing the proposed constructs and relationships against the researchers’ conceptual framework [41,45]. Furthermore, the interrelation among the first-order latent constructs was analyzed using full structural model including the path analysis [46]. The paths were determined utilizing literature review, expert consultations, co-design meetings with the *hijra* community, and understanding of the research domain. The models were fitted using maximum likelihood estimation to account for all available data. Statistical significance was determined at the p < 0.05 level.

Evidence-based estimated standardized regression weights, also referred to as factor loading (FL),was used to express the results derived from SEM, where the threshold was considered above 0.7. This signified that the indicator provided adequate value to explain the unobservable construct [41][href:.]. Model fits were evaluated using the Chi-sqr/df ratio, required to be less than 3, the Standardized Root Mean Square Residual (SRMR), which was expected to be between 0.05 and 0.09, the Root Mean Square Error of Approximation (RMSEA). This was expected to be less than or equal 0.10. The Tucker Lewis Index (TLI), which was expected to be greater than 0.95, and Comparative Fit Index (CFI), which was also expected to be greater than 0.95 [47,48]. Factor loading (FL) over 0.7 was considered to add proper value in understanding the unobserved constructs [41]. Modification indices were not used in this study as there was an adequate model fit. Additionally, univariate analysis was performed to describe the frequency distribution of the variables and further contextualize the results. All statistical analyses in this study were conducted using several software, namely SPSS version-27 and AMOS version-26.

### Ethical considerations

The study proposal was reviewed and approved by the Institutional Review Board (IRB) of icddr,b, i.e., the Research Review Committee and Ethical Review Committee, following all the relevant ethical guidelines, such as the Declaration of Helsinki. Verbal informed consent was taken from the study participants after explaining the objectives, benefits, and risks of the research. We needed to take verbal consent because many of the *hijra* are often involved with sexual behaviors beyond legalized heterosexual intercourse. Therefore, they often refuse to provide written consent due to fear of identity disclosure and potential legal repercussions. Participants were provided with the right to decline answering any question or opting out of the study. They were also ensured that declining or withdrawing from participation would have no effect whatsoever on the treatment and services they receive from the existing HIV prevention service centers. Notably, after completing the survey, participants were also given thank-you payments in the form of conveyance allowances, following the ethical principles of the Institutional Review Board of icddr,b.

## Results

Findings showed that various social categories such as gender identity, sexual orientation, occupation, socioeconomic status and (financial) ability, coupled with connected systems and structures of power, influenced their structural inequalities and worsened their healthcare seeking behaviors. These inequalities and healthcare-seeking behaviors were reflected in the analysis of the structural equation modelling exercise. Based on this, **Fig 3** illustrates the distribution of healthcare access barriers among the *hijra* participants, comprising four primary themes: Harassment, Financial difficulties, Discrimination, and Lack of infrastructural facilities. According to **Fig 3**, all *hijra* included in the study experienced at least one or more healthcare access barrier. The percentage of individuals facing only a single healthcare access barrier was notably low, at only 3.7%.

Conversely, the highest percentage (53.5%) of *hijra* participants encountered all four healthcare access barriers. This shows the influence of multiple forms of oppression, clustering of interrelated barriers, and existing power structures on their healthcare access.

### Structural equation modeling (SEM)

**Fig 4:**
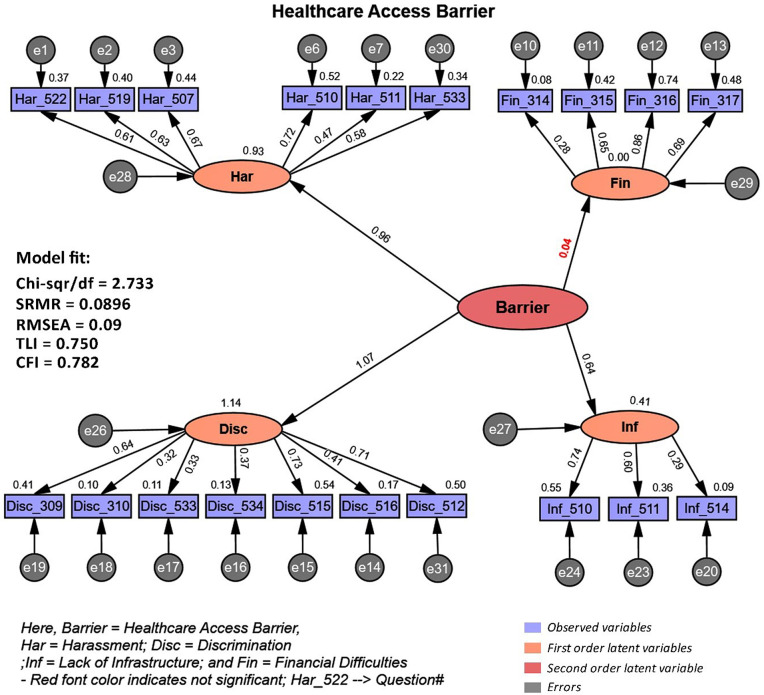
Fitted structural equation model based on standard values on healthcare access barrier.

According to **Fig 4** and **Table 2**, harassment, discrimination, and lack of infrastructural facilities were significantly related to healthcare access barriers, except for financial difficulties. Observing the standardized estimates, harassment, discrimination, and lack of infrastructural facilities had a visibly positive relationship with healthcare access barriers. This indicates that increased harassment, discrimination, and lack of infrastructure would increase healthcare access barriers for the *hijra* community.

**Table 2:** Estimates and goodness of fit of the second order model (results from SEM).

Component	Path	Construct	Est.	SE	CR	P value	Std. Est.
Financial difficulties	←	Healthcare access barrier	0.02	0.05	0.41	0.68	0.04
Lack of infrastructural facilities	←	Healthcare access barrier	0.28	0.10	2.99	**	0.64
Discrimination	←	Healthcare access barrier	1.00				1.07
Harassment	←	Healthcare access barrier	1.47	0.30	4.88	***	0.96
**Har_522:** Ever faced verbal harassment in medical settings	←	Harassment	0.86	0.11	7.62	***	0.61
**Har_519:** Negative attitude of healthcare providers during last visit	←	Harassment	1.00				0.63
**Har_507:** Negative response from service providers while revealing *hijra* identity at public healthcare facilities	←	Harassment	1.37	0.17	8.14	***	0.67
**Har_510:** Entry level harassment at public healthcare settings	←	Harassment	1.18	0.14	8.61	***	0.72
**Har_511:** Entry level harassment at private healthcare settings	←	Harassment	0.56	0.09	5.98	***	0.47
**Har_533:** Types of harassment experienced at healthcare facilities	←	Harassment	0.64	0.09	7.35	***	0.58
**Fin_314:** Lack of emergency healthcare savings	←	Financial difficulties	1.00				0.28
**Fin_315:** Financial difficulties to receive healthcare services ever in life-time	←	Financial difficulties	2.28	0.62	3.66	***	0.65
**Fin_316:** Expenditure tradeoff to seek healthcare services ever in life-time	**←**	Financial difficulties	1.41	0.42	3.36	***	0.86
**Fin_317:** Disruption/ abstinence from treatment due to lack of money in the last one year	**←**	Financial difficulties	0.91	0.26	3.47	***	0.69
**Disc_516:** Negligence from private healthcare providers while seeking treatment	**←**	Discrimination	1.00				0.41
**Disc_515:** Negligence from public healthcare providers while seeking treatment	**←**	Discrimination	2.32	0.41	5.67	***	0.74
**Disc_534:** Refusal/ denial of treatment by the health care providers while seeking treatment ever in life-time	**←**	Discrimination	0.26	0.06	4.03	***	0.37
**Disc_533:** Types of discrimination experienced at health care facilities ever in life-time	**←**	Discrimination	0.29	0.08	3.78	***	0.33
**Disc_310:** Feelings of discomfort while sharing health problems with healthcare professionals at private healthcare facilities	**←**	Discrimination	0.52	0.14	3.76	***	0.32
**Disc_309:** Feelings of discomfort while sharing health problems with healthcare professionals at public healthcare facilities	**←**	Discrimination	1.22	0.23	5.37	***	0.64
**Disc_512:** Delayed access to treatment compared to general population in the last one year	**←**	Discrimination	2.43	0.43	5.69	***	0.71
**Inf_514:** Healthcare facilities’ distanced location to get healthcare services	**←**	Lack of infrastructural facilities	1.00				0.29
**Inf_511:** Lack of infrastructural facilities in private hospitals	**←**	Lack of infrastructural facilities	2.38	0.69	3.46	***	0.60
**Inf_510:** Lack of infrastructural facilities in public hospitals	**←**	Lack of infrastructural facilities	3.55	1.07	3.33	***	0.74
**Chi-sqr./ df = 2.733**							
**SRMR = 0.0896**							
**RMSEA = 0.09**							
**TLI = 0.750**							
**CFI = 0.782**							

Here, ** significant at p < 0.01, *** significant at p < 0.001; Est. = Estimate; SE = Standard Error; CR = Critical Ratio; Std. Est. = Standard Estimate; Corr. = Squared multiple correlation.

Among the three first-order latent constructs, discrimination was the most strongly associated with healthcare access barriers, harassment, and lack of infrastructural facilities.

Negligence from public healthcare providers while seeking treatment (Disc_515), i.e., problems while consulting a public hospital physician/ healthcare provider, such as not listening to health problems or not properly inquiring about the issues, were strongly and significantly related (FL = 0.74) to discrimination. This indicates that negligence from public healthcare providers based on their *hijra* identity while seeking treatment increased discrimination and incurred healthcare access barriers for this community. However, this type of negligence from private healthcare providers was seen to be significantly related to discrimination, but with an insufficient FL. Other indicators such as denying treatment, refusing to examine body parts due to their gender identity and sexual orientation, and feeling of discomfort due to the perception of discrimination while sharing health problems in both public and private healthcare facilities increased the latent construct of discrimination. Although these indicators demonstrate a significant association with discrimination, their FL was inadequate.

In contrast, discrimination in the form of delayed access to treatment compared to the general population (Disc_512) due to their gender identity and sexual orientation, i.e., experiencing significant delays compared to others in accessing medicines, treatment intervention, such as surgery or other procedures, diagnostic tests, support from a nurse, an appointment with a primary care doctor, or an appointment with a specialist had strong (FL = 0.71) relation to discrimination. This indicates that delayed access to treatment compared to the general population based on their gender and sexual orientation syndemically increased discrimination and ultimately posed healthcare access barriers for the *hijra* community within the last year.

In further analyzing **Fig 4** and **Table 2**, within the first order latent construct, entry-level harassment at public healthcare settings (Har_510), i.e., facing problems such as entry barriers through hospital gates, problems with standing in a queue, problems with entering their names, and purchasing tickets had the highest factor loading (FL = 0.72) related to harassment. All of these harassment-related barriers are rooted in their gender identities, sexual orientations and socioeconomic positioning. On the other hand, entry-level harassment at private healthcare was also significantly related to harassment, although the FL was not adequate in this case. Similarly, other harassment indicators, such as instances of verbal abuse in healthcare facilities, negative attitudes from healthcare providers during previous visits, and negative responses or misbehavior in public healthcare facilities, were also identified as significantly related to harassment. Additionally, various forms of harassment, such as belittling or ridiculing for being a *hijra* or the rejection of their gender identity amongst healthcare providers, were found to be significantly linked to harassment, thus ultimately creating healthcare access barriers. However, the available evidence for these associations falls below the threshold of FL = 0.7, indicating inadequate strength of support for the observed relationships.

In the case of lack of infrastructural facilities (Inf_510), i.e., lack of separate toilets and beds for *hijra* in public healthcare facilities were the most strongly associated with (FL = 0.74) the lack of infrastructural facilities. Similarly, the lack of infrastructural facilities in private hospitals and the distance from healthcare services were also significant in relation to the lack of infrastructural facilities. However, the FL fell below the threshold of 0.7.

All of the mentioned factors relate to healthcare access barriers through their respective first-order latent construct, i.e., harassment, discrimination, and lack of infrastructural facilities. In general, it was observed that all explored indicators within harassment, discrimination, and lack of infrastructural facilities significantly contributed to the construction of increased barriers. The model was found to fit according to Chi-sqr/df = 2.733, SRMR = 0.0896, and RMSEA = 0.090 criteria, where Chi-sqr/df less than 3, and the SRMR value between 0.05 and 0.09, RMSEA value less than 0.10 indicates adequate model fit [47,48].

**Fig 5:**
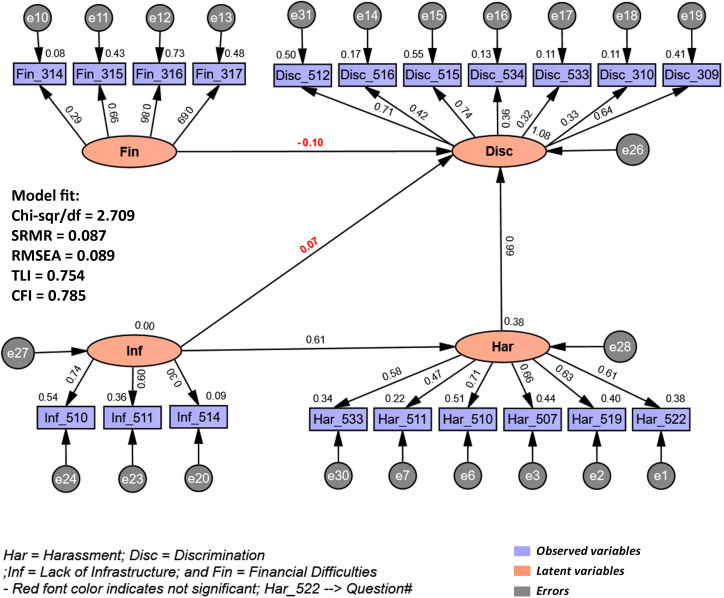
Fitted structural equation model based on standard values of the first order model.

Further analysis was conducted within the first order latent variables to examine the interrelationship among the first order latent constructs with full structural equation modeling, including path analysis. The results were depicted in Fig 5 and Table 3. These results indicate that, within the first order constructs, lack of infrastructure was significantly related (FL = 0.61) to harassment, and harassment was ultimately significantly related (FL = 0.99) to discrimination. Although lack of infrastructural facilities was not significantly related to discrimination, it has effects such as influencing harassment and then leads towards discrimination. Financial difficulties were also seen to have no significant relation with the other latent constructs, although it showed the influence of *hijra*’s socioeconomic class, occupation, and lack of financial ability, and these circumstances’ relationship with one another.

**Table 3:** Estimates and goodness of fit of the first order model (results from SEM).

Component	Path	Construct	Est.	SE	CR	P value	Std. Est.
Harassment	←	Lack of infrastructural facilities	2.12	0.67	3.16	**	0.61
Discrimination	←	Harassment	0.62	0.12	5.14	***	0.99
Discrimination	←	Financial difficulties	−0.16	0.09	−1.74	0.082	−0.10
Discrimination	←	Lack of infrastructural facilities	0.16	0.21	0.75	0.451	0.07
**Chi-sqr/df = 2.709**							
**SRMR = 0.087**							
**RMSEA = 0.089**							
**TLI = 0.754**							
**CFI = 0.785**							

Here, ** significant at p < 0.01, *** significant at p < 0.001; Est. = Estimate; SE = Standard Error; CR = Critical Ratio; Std. Est. = Standard Estimate.

In this case, the model was also found to fit according to Chi-sqr/df = 2.709, SRMR = 0.087, and RMSEA = 0.089 criteria, where Chi-sqr/df less than 3, and the SRMR value between 0.05 and 0.09, RMSEA value less than 0.10 indicates adequate model fit [55,56].

## Discussion

**Fig 6:**
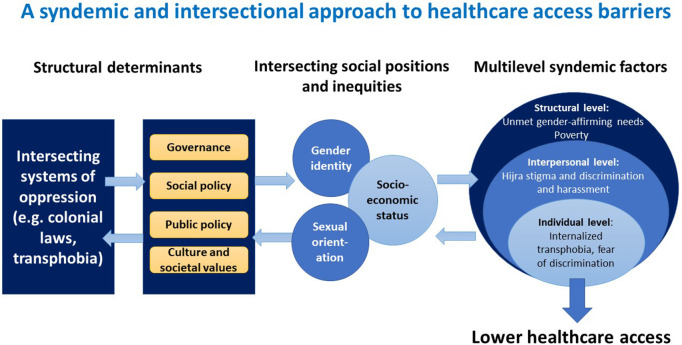
A syndemic and intersectional approach to healthcare access barriers among *hijra.*

This is the first study in Bangladesh and its region to employ structural equation modeling to explore and understand healthcare access barriers of *hijra*, particularly through intersectionality [37]. We applied intersectionality to examine how multiple interlocking identities such as gender, sexual orientation, occupation, socioeconomic class and lack of financial ability interacted with power systems and structures (e.g., policies and laws regarding *hijra*, homosexuality and healthcare provisions for *hijra*) to displace *hijra* towards structural inequalities in the healthcare system (i.e., healthcare access barriers). Moreover, syndemic theory was used to understand how multiple structural and psychosocial factors cluster and reinforce one another. Recent scholarship has emphasized that syndemics are not merely limited to biomedical conditions, but also embody institutional and structural challenges, namely discrimination, stigma and infrastructural limitations, all of which cluster and reinforce one another amongst marginalized groups [35]. By blending two theories, this study illuminated why hijra are structurally positioned to experience healthcare access barriers (**Fig 6**).

The findings revealed that systemic and institutionalized discrimination of *hijra* within healthcare settings often displaced *hijra* at an unfair advantage, coupled with their gender identity and sexual orientation. This led to 54% of the *hijra* experiencing four types of barriers, including discrimination, harassment, lack of infrastructural facilities, and financial constraints to access healthcare. Our SEM illustrated that harassment, discrimination, and lack of infrastructural facilities were significantly associated with healthcare access barriers except for financial difficulties (**Fig 4** and **Table 2**). Notably, discrimination, harassment, infrastructural conditions, and financial limitations were not isolated barriers, rather they embodied interacting structural conditions. These barriers have co-occurred, clustered and compounded due to shared structural determinants such as stigma, gender regulation, and institutional neglect. As 54% have experienced all four barriers, this indicates the presence of multiple concurrent barriers which are strongly interrelated.

Our model further indicated that discrimination by healthcare providers was the most significant barrier (FL: 1.07) to healthcare access among *hijra*, followed by harassment and lack of infrastructural facilities. All of these structural inequalities were linked to their non-conforming gender identity, sexual orientation, and compromised social position along with the infrastructural and health workforce limitations of the hospital setting, all of which are rooted in limited institutionalized acceptance of *hijra* [49]. From a syndemic perspective, discrimination was a key driver that intensified other barriers, such as harassment and infrastructural limitations. Discrimination could potentially legitimize mistreatment by healthcare providers and health support staff, aggravate discomfort in gender-segregated spaces, and impede healthcare-seeking behaviors. This magnifies the cumulative burden of healthcare exclusion.

Similarly, a local review article [16] also highlighted the pervasiveness of discrimination in healthcare settings in Bangladesh faced by this population, particularly in terms of their testing and treatment uptake abilities based on their gender identity. This is rooted in the lack of knowledge and understanding about gender diversity, as a result of an upbringing that has rigidly engrained gender binary ideologies. Therefore, they are unable to accept the concept of any identity beyond this binary [24]. Moreover, since heterosexual behaviors are considered the norm within Bangladesh’s socio-cultural context, same-sex behaviors also characterize a source of oppression for *hijra* [49]. Consequently, this attitude has been transposed in institutions, including healthcare settings, where *hijra* are not considered as equally entitled as their cisgender counterparts [49]. Similarly, other literature based in Bangladesh have reflected that the same socio-cultural barriers negatively impacted their ability to seek healthcare [50].

These accounts of discrimination among transgender populations, which are also produced by oppressive systems of power, were also corroborated by studies based in India and South America [9,50]. Specifically, transgender populations in India experience discrimination across various healthcare settings, particularly in the uptake services for HIV testing, antiretroviral therapy and sexual health services [24]. Additionally, a study in Guatemala revealed adverse experience in the healthcare setting including mockery, ignorance of patient confidentiality, and refusal of care, some of which were also found in our study [51]. On the other hand, our findings further delineated the settings and nature of discrimination (i.e., negligence, refusal, feelings of discomfort to share information, and delayed treatment access), which other studies did not explore to the same degree. Moreover, our findings linked these relationships to the *hijra*’s multiple social identities and overarching structural barriers which could predispose them to discrimination. For instance, unwillingness to listen to health problems and incomplete history taking of *hijra* predominantly by the physicians at public health facilities were found to contribute to discrimination. However, listening to complete health complaints constitutes the core of proper patient history-taking.

Our study indicated that *hijra* encountered multifaceted degrees of harassment (i.e., the second largest barrier as per the SEM) in accessing healthcare services, such as impediments to entering the hospital and standing in the queue, challenges in entering their names, and difficulties in purchasing the ticket. All of these impediments were found to be a product of an oppressive power differentials towards *hijra*, where their gender is not recognized, thus imbuing structural inequalities. This finding about harassment was also documented by a US-based study, though the multifaceted exploration of that study was limited [52]. Moreover, a case-based scoping review that covered some of the healthcare access challenges of transgender populations corroborated this phenomenon, citing that most transgender populations had to endure misbehavior from culturally incompetent healthcare providers [53]. Whereas, our study supplemented that information by revealing the magnitude of the harassment on a quantifiable scale and linking it to the aggregate effect of both their gender identity and sexual orientation. Infrastructural barriers were identified as another significant healthcare access barrier, albeit not to the same degree as discrimination and harassment. Many of these *hijra* expressed feeling uncomfortable with public healthcare services merely because of their gender identity and sexual orientation. These feelings of discomfort were also reflected in a Brazil-based study on transgender women [8]. These adversities were also found at the regional level, where a scoping review in India identified discrimination as a key barrier in the healthcare setting [24]. Whereas, our study further delineated the specifics of the infrastructural barriers and linked them to their gender identity and sexual orientations, and the oppressive power systems of the healthcare setting.

Moreover, our study findings demonstrated financial barriers as one of the healthcare access barriers nestled in the conceptual framework, though not statistically significant as per the SEM. This is attributed to other sources of income generation among *hijra* such as *cholla* and *badhai* [13,49]. However, our analysis identified an adequate fit of the modelling of healthcare access barriers (see Table 3). Based on the intersectionality theory, this demonstrates that their socioeconomic class, occupation and lack of financial ability (despite being statistically insignificant) negatively influenced their access to healthcare. Moreover, the healthcare system is designed in such as way that there are no discounts or other equitable measures to place *hijra* on the forefront of healthcare. Though financial limitations were not statistically significant in the structural equation model, a syndemic perspective indicates that economic vulnerability is embedded within a broader umbrella of structural challenges experienced by *hijra*. Income-generating activities such as *badhai* and *cholla* could buffer some direct healthcare costs, but would not alleviate the syndemic effects of harassment, discrimination and infrastructural limitations, which can collectively reduce healthcare access. Similarly, other literature across the globe substantiated this finding, claiming that the lack of insurance coverage and the inability to pay out of pocket compromised their healthcare-seeking behaviors [54–56]. However, these studies underpinned the statistical significance of financial constraints in terms of their ability to uptake healthcare services.

Together, our study demonstrated that intersecting identities and structural power relations cultivated a syndemic pattern of healthcare exclusion. While intersectionality illustrated the structural positioning of *hijra* within healthcare systems, syndemic theory showed that multiple, interconnected barriers clustered and reinforced each other within healthcare institutions. This integrated framework highlights the need for interventions that transcend single barriers, thus underscoring the need for systems-level reforms.

### Strengths

This study was conducted for the first time among *hijra* to explore health-seeking behaviors and healthcare access barriers in 16 districts throughout eight divisions in Bangladesh. This research opted for SEM as a statistical method due to its capacity to accommodate complex modelling concepts [41,45,47]. In our study, SEM enabled the examination of latent variables, particularly healthcare access barriers experienced by the *hijra* community,. As this cannot be feasibly explored using conventional regression models or similar statistical techniques, this illuminates the superiority of SEM over other statistical models [52,53,55]. It also supersedes other statistical models as it is confirmatory, allowing us to confirm our conceptual understanding of the mechanisms underlying healthcare access barriers faced by *hijra* communities (see **Fig 1**) [41,45,47].

### Limitations

As behavioral information was collected through face-to-face interviews, this opened avenues for recall and social desirability biases. Furthermore, due to the limited observations, it was not possible to conduct an analysis of multiple groups in relation to different socio-demographic factors. For the same reason, a separate sub-sampled test for reliability and validity of the overall model was not possible. Notably, due to the sensitive nature of the issues, we could not account for legal and identity barriers as part of the analysis. This is because of various structural circumstances which emerged prior to the study period. Specifically, since Bangladesh is a predominantly conservative society, ideologies beyond the gender binary are socially unaccepted. Moreover, anti-transgender rhetoric and murders for rights activism escalated within the past few years, thus cultivating climates of fear and panic among *hijra*. As a result, questions about legal and identity barriers incurred apprehension and discomfort during field testing, thus leading us to exclude this construct.

It is worth mentioning that, in our study, we have investigated healthcare access barriers, which are not directly observable variables. Instead, it is a second-order latent construct, where a second-order latent construct indicates a higher-order factor combining two or more first-order latent constructs to represent an abstract and overarching construct. Thus, full SEM in the second order modeling was out of the scope of this study. However, the interrelations and paths were analyzed within the first order modeling of this study.

### Implication of findings and recommendations

Our study findings provided crucial evidence and directives for improving health infrastructure to promote gender inclusivity toward *hijra* and other transgender populations worldwide. The analysis from the structural equation modelling showed the magnitude of discrimination, harassment and infrastructural limitations in the healthcare setting, demonstrating the interplay between clustering socio-structural barriers faced by *hijra* and their reported experiences in the healthcare facilities. This pattern also resonates with the intersectionality and syndemic theories. In addition, quantitative data have provided insights into the priority areas that could better address their unmet healthcare needs. These insights could be applied to healthcare settings in Bangladesh and similar locations worldwide where *hijra* and other transgender populations experience healthcare access barriers due to gender identity and sexual orientation. Since discrimination was identified as the most significant barrier as per the SEM, specific initiatives could be adopted to address discrimination. For instance, discrimination could be addressed at various levels where *hijra* are mobilized towards healthcare through involving *hijra* in healthcare system delivery programs. For example, a recent scoping review demonstrated promising outcomes of several quasi-experimental studies and clinical trials of peer-based interventions, in terms of increasing healthcare uptake [57]. Similarly, another recent qualitative study indicated that leveraging peer support is crucial for promoting healthcare uptake across diverse transgender communities, as it can increase connectivity, collectivize marginalized populations, and share experiences [58].

This could be integrated with collaborative learning opportunities with *hijra* communities, family members and healthcare providers [59]. Moreover, the treatment rights of *hijra* must be included in the citizen charter and framed on the hospital wall to increase awareness about *hijra* rights among healthcare providers, support staff and other patients. The government needs to also pass an anti-discriminatory law to allow *hijra* to exercise their rights against gender-based discrimination at healthcare facilities. The Thailand government passed a similar law, titled the Gender Equality Act (2015). Similarly, in Australia, under the Sex Discrimination Act, it is against the law to discriminate based on gender identity in any setting. Within the broader South Asian region, India’s Transgender Persons (Protection of Rights) Act 2019 underscored the protection of transgender persons from discrimination, recognizing their gender identity, and providing welfare support for education, healthcare and legal identity recognition [60]. Given socio-cultural similarities with Bangladesh, such protection laws could be subsequently established.

Moreover, like neighboring countries such as India [61] and Pakistan [62], laws and rights protection acts need to be formed to ensure healthcare settings are as inclusive and equitable towards *hijra* as other genders, and their gender identity and sexual orientation do not subject them to unfair disadvantage. Similarly, it would be beneficial to develop a health service delivery guideline for *hijra* based on international guidelines, e.g., The World Professional Association for Transgender Health [63] and the United Nations Development Program. However, this needs to be adapted according to the local context.

Additionally, at the policy level, *hijra*-inclusive health policies could be considered through the participation of *hijra* community leaders and other stakeholders, similar to other Southeast Asian countries such as Thailand, Indonesia and Philippines [59]. Moreover, efforts need to be introduced to integrate culturally competent healthcare within the healthcare setting. For instance, the Pehchan program in Tamil Nadu, India, a multifaceted empowerment program, worked with the local government to provide social entitlements, documents and amenities such as a ration card, an access card for public hospitals a social welfare card for people living below the poverty line [58]. Similarly, the Asia Pacific Transgender Network facilitated a trans-inclusive healthcare workshop in Nepal where they collaborated with local transgender advocacy groups to develop training modules for healthcare providers [64]. For this, evidence-based approaches could be applied such as gender-inclusive health forms (i.e., male, female, and *hijra*/others); separate infrastructural facilities for *hijra* including separate beds, wards, restrooms, and queues for *hijra* to absolve them of the pressure of conforming to the gender binary.

Transgender-related health issues need to be incorporated into medical school curriculum. In addition, healthcare providers (including physicians, nurses, and medical technologists) need to be trained on sexual health and rights of *hijra*, gender identity, sexuality of *hijra*, etc., before being onboarded to work in the healthcare sector. More awareness programs on the hardships of *hijra* life, health needs, and problems need to be publicized in electronic and print media to promote gender inclusivity across various platforms. Moreover, access to quality healthcare could be expanded for *hijra* using the informed consent model, which could be monitored and evaluated through supplementary research capturing voices from the *hijra* community. Additionally, since *hijra gurus* often play a pivotal role in the healthcare decision-making aspects of the *hijra* community, the *gurus* could also be sensitized and mobilized towards seeking healthcare.

## Conclusion

Our study findings revealed that a considerable portion of the *hijra* throughout various urban and rural districts across Bangladesh struggle with harassment, discrimination, financial constraints, and lack of infrastructural provisions as the most pressing healthcare access barriers. The structural equation modelling analysis indicated that the most significant barriers included discrimination and harassment by healthcare providers, followed by the lack of tailored infrastructure to cater to the *hijra*’s health needs. These barriers signify that despite being acknowledged as a separate gender category within the existing governmental framework, they remain subjected to multiple intersecting forms of oppression which influence their ability to seek viable healthcare. In this context, healthcare facilities have lengths to go until universal health coverage can be achieved, particularly among marginalized, stigmatized populations. However, to fully realize universal health coverage, it is integral to adopt appropriate, context-specific measures to eliminate harassment and discrimination, whilst also improving infrastructural facilities. While this study was able to explore the most concerning healthcare access barriers, some issues warrant further exploration. Specifically, complementary research could explore individual behaviors and decision-making processes influencing healthcare-seeking, alongside examining the effect of other variables, e.g., sociodemographic characteristics, in relation to the SEM.

## Supporting information

S1 File Inclusivity-in-global-research-questionnaire. (DOCX)
